# Is Hippocampal Resection Necessary for Low-Grade Epilepsy-Associated Tumors in the Temporal Lobe?

**DOI:** 10.3390/brainsci12101381

**Published:** 2022-10-12

**Authors:** Yutaro Takayama, Naoki Ikegaya, Keiya Iijima, Yuiko Kimura, Kenzo Kosugi, Suguru Yokosako, Yuu Kaneko, Tetsuya Yamamoto, Masaki Iwasaki

**Affiliations:** 1 Department of Neurosurgery, National Center Hospital, National Center of Neurology and Psychiatry, Kodaira 187-8551, Tokyo, Japan; 2 Department of Neurosurgery, Graduate School of Medicine, Yokohama City University, Yokohama 236-0004, Kanagawa, Japan

**Keywords:** hippocampus, low-grade epilepsy-associated tumors, temporal lobe, verbal function

## Abstract

Low-grade epilepsy-associated tumors (LEATs) are common in the temporal lobe and can cause drug-resistant epilepsy. Complete resection of LEATs is sufficient for seizure relief. However, hippocampal resection might result in postoperative cognitive impairment. This study aimed to clarify the necessity of hippocampal resection for seizure and cognitive outcomes in patients with temporal lobe LEATs and a normal hippocampus. The study included 32 patients with temporal lobe LEATs and without hippocampal abnormalities. All patients underwent gross total resection as treatment for drug-resistant epilepsy at our tertiary epilepsy center from 2005 to 2020, followed by at least a 12-month follow-up period. Seizure and cognitive outcomes were compared between patients who underwent additional hippocampal resection (Resected group) and those who did not (Preserved group). Among the participants, 14 underwent additional hippocampal resection and 28 (87.5%) achieved seizure freedom irrespective of hippocampal resection. The seizure-free periods were not different between the two groups. Additional hippocampal resection resulted in a significantly negative impact on the postoperative verbal index. In conclusion, additional hippocampal resection in patients with temporal lobe LEATs without hippocampal abnormalities is unnecessary because lesionectomy alone results in good seizure control. Additional hippocampal resection may instead adversely affect the postoperative language function.

## 1. Introduction

Low-grade epilepsy-associated tumors (LEATs) are the primary causes of drug-resistant epilepsy, particularly in children and young adults. LEATs are known to be slow-growing and biologically benign [1], although a few cases with tumor progression or malignant transformation have been reported [2,3]. LEATs account for 2–5% of brain tumors and are the second most common etiology of surgically amenable epilepsy. The tumors arise most commonly in the temporal lobe; hence, they may cause drug-resistant limbic seizures. Complete resection of the tumor is generally sufficient for seizure relief [4,5,6]. Tumor invasion of the hippocampus or hippocampal sclerosis can be associated with temporal LEATs. In such cases, resection of the hippocampus may be considered for seizure control.

Additional hippocampal resection results in a higher likelihood of seizure resolution than lesionectomy alone in patients with World Health Organization (WHO) grade II gliomas [7]. WHO grade I tumors, often diagnosed as ganglioglioma or dysembryoplastic neuroepithelial tumors, account for more than 65% of LEATs [8]. When tumor invasion or sclerotic changes are absent in the hippocampus, additional hippocampal resection may result in postoperative cognitive impairment. The benefit of additional hippocampal resection at the expense of cognitive impairment has not been clarified, as very few studies that evaluated its effects on cognitive function included patients with a normal hippocampus. We hypothesized that tumor resection alone would provide adequate seizure control in patients with a normal hippocampus and that additional resection of the normal hippocampus could affect the postoperative cognitive status.

This study aimed to clarify the necessity of hippocampal resection in patients with temporal lobe LEATs without hippocampal abnormalities to evaluate our hypothesis and patient outcomes regarding seizure control and cognitive function.

## 2. Materials and Methods

Clinical information of the patients and their history of epilepsy, including the tumor location, presence of a hippocampal abnormality, extent of resection, pathological diagnosis, and postoperative seizure and cognitive outcomes, were collected retrospectively from our database based on medical records. We compared the seizure and cognitive outcomes between patients who underwent additional hippocampal resection and those who did not undergo the procedure. The study was approved by the ethics committee of the National Center of Neurology and Psychiatry, Tokyo, Japan (No. A2018-049), and the requirement for written informed consent was waived because of the study’s retrospective design.

### 2.1. Patients

The inclusion criteria were as follows: (1) consecutive patients with temporal lobe LEATs who underwent epilepsy surgery at our tertiary epilepsy center from 2005 to 2020; (2) absence of hippocampal abnormality, including hippocampal sclerosis and hippocampal invasion of the tumor, on magnetic resonance imaging (MRI); (3) underwent gross total resection (GTR) of the tumor; and (4) followed up for at least 12 months after resection. A total of 56 patients underwent epilepsy surgery for temporal lobe LEATs during the study period. Among them, 1 patient underwent two-stage hippocampal resection after tumor resection, 21 patients had hippocampal lesions detected by MRI, and 2 patients who failed to achieve GTR were excluded. Finally, 32 patients (13 females) were included in the analysis. Our board-certified pathologists pathologically confirmed the diagnosis of LEATs.

### 2.2. Presurgical Evaluation

All patients underwent comprehensive epilepsy evaluations, including medical interviews, neurological and neuropsychological examinations, long-term video-electroencephalography (EEG), and MRI. Epilepsy histories were obtained from all patients and, when necessary, from their relatives, and the semiology of their habitual seizures was confirmed. Scalp EEG was recorded with the standard 10–20 electrode placement system, including bilateral anterior temporal electrodes. Three-Tesla brain MRI was performed, including three-dimensional fluid-attenuated inversion recovery, double inversion recovery, and T1- and T2-weighted imaging, with contrast-enhanced MRI performed when necessary. We evaluated the location of LEATs and the presence of abnormalities in the hippocampus. Based on the comprehensive epilepsy evaluations, the LEATs were suspected as epileptogenic in all patients, and the institutional patient-management conference determined an indication for surgical resection.

### 2.3. Surgical Resection

GTR of the MRI lesion (tumor) was attempted in principle. The decision to perform hippocampal resection was usually made in advance; occasionally, it was made intraoperatively based on the electrocorticography (ECoG) findings. The hippocampus was defined as preserved when hippocampal resection was not performed, and postoperative MRI confirmed that the hippocampal head was preserved (Preserved group); otherwise, the hippocampus was considered resected (Resected group). The reasons for preserving the hippocampus were then investigated.

### 2.4. Postoperative Seizure Outcome

The postoperative seizure outcome was assessed during the final follow-up based on the International League Against Epilepsy (ILAE) classification [9]. The seizure-free rate was statistically compared between the Resected and Preserved groups using Fisher’s exact test (JMP version 11, SAS Institute Inc., Cary, North Carolina). The seizure-free period (SFP), defined as the seizure-free duration after surgery until the first seizure recurrence, was analyzed using the Kaplan–Meier method, and the differences in the median SFP between the two groups were assessed using the log-rank and Wilcoxon tests (R version 3.5.2, The R Foundation for Statistical Computing, Vienna, Austria). A *p*-value < 0.05 was considered statistically significant.

### 2.5. Antiseizure Medication (ASM) Reduction

The numbers of ASMs at the time of surgery and the final follow-up were compared. The association between hippocampal resection and successful ASM withdrawal after surgery was statistically evaluated.

### 2.6. Postoperative Cognitive Outcome

Neuropsychological evaluations were performed pre- and postoperatively. The intelligence quotient (IQ) was measured using the Wechsler Adult Intelligence Scale (WAIS), Wechsler Intelligence Scale for Children (WISC), or Suzuki–Binet test [10]. The developmental quotient (DQ) was measured in young children by the Kinder Infant Developmental Scale (KIDS) [11] or Enjoji test [12]. Both pre- and postoperative assessments used the same tests.

Cognitive function was categorized into three components: global index (GI), verbal index (VI), and working memory index (WMI). The GI was represented by the full-scale IQ in the WAIS or WISC or by the standardized IQ/DQ when evaluated with other scales. The standardization of IQ/DQ was performed using the following formula:

GI = 10 × (x − μ)/σ + 100 (x: measured score; μ: average score; σ: standard deviation)

The average score (μ) was referenced from previous reports: 111 in the Suzuki–Binet test and 105 in the KIDS and Enjoji test [10,11,12,13].

The VI was represented by the verbal comprehension score in the WAIS or WISC or by the standardized value of the averaged sub-scores on language comprehension and language expression in KIDS [14]. The WMI was represented by the working memory score in the WAIS and WISC.

The pre- and postoperative indices between the Resected and Preserved groups were statistically compared using Student’s *t*-tests. The differences between the pre- and postoperative indices were statistically analyzed using the paired *t*-test. The preoperative cognitive indices and effects of hippocampal resection on the postoperative cognitive indices were analyzed using analysis of covariance (ANCOVA). All statistical analyses were performed using JMP software (version 11, SAS Institute Inc., Cary, North Carolina). A *p*-value < 0.05 was considered significant.

## 3. Results

### 3.1. Patient and Tumor Characteristics

The clinical characteristics of the 32 patients (32 tumors) are summarized in Table 1.

The mean ages at initial epileptic seizure and surgery were 8.1 (range, 0–18) and 18.6 (range, 0.5–55) years, respectively. The mean duration of postsurgical follow-up was 67.7 (range, 12–170) months. Among the patients, 75.0% were right-handed; handedness was not determined in 15.6% of the patients, mainly because of their young age. The most common seizure type was focal onset impaired awareness seizures (96.9%). Seizure frequency was daily in 3 cases, weekly in 15 cases, monthly in 10 cases, and rare in 4 cases.

**Figure 1:**
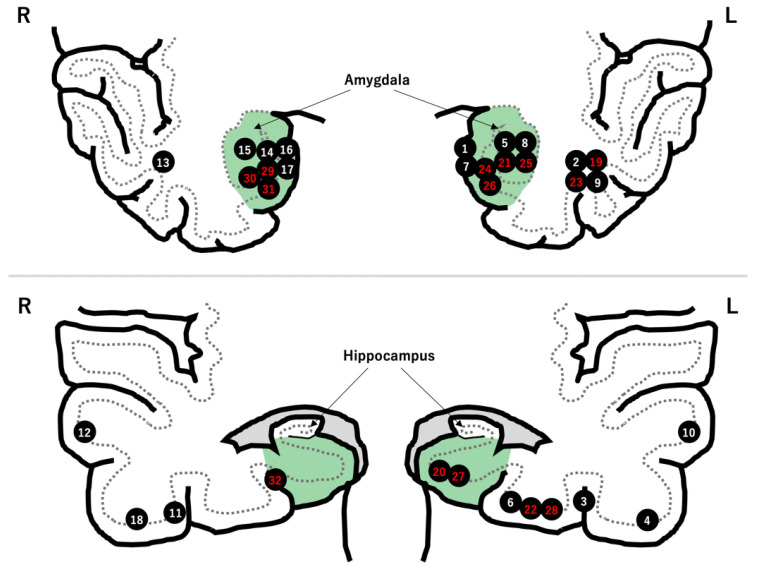
Locations of the 32 LEATs. There were 18 tumors in the mesial temporal region (green zone). The patient number is highlighted in red after hippocampal resection.

The tumors were in the left temporal lobe and medial to the collateral sulcus in 20 (62.5%) and 18 (56.3%) patients, respectively (Figure 1). Cystic formation and calcification were seen in 22 (68.8%) and 13 (40.6%) tumors, respectively. The pathological diagnosis was ganglioglioma in 15 patients (45.6%), low-grade glioma or astrocytoma in 15 patients, glioneuronal tumor in 1 patient, and dysembryoplastic neuroepithelial tumor in 1 patient (Table 2).

Focal cortical dysplasia was seen adjacent to the tumor in two patients with low-grade glioma. No patients underwent chemo/radiotherapies after surgery or showed tumor recurrence.

### 3.2. Hippocampal Resection

Fourteen patients underwent hippocampal resection. Of these, 10 patients had tumors located medial to the collateral sulcus (Figure 1).

**Figure 2:**
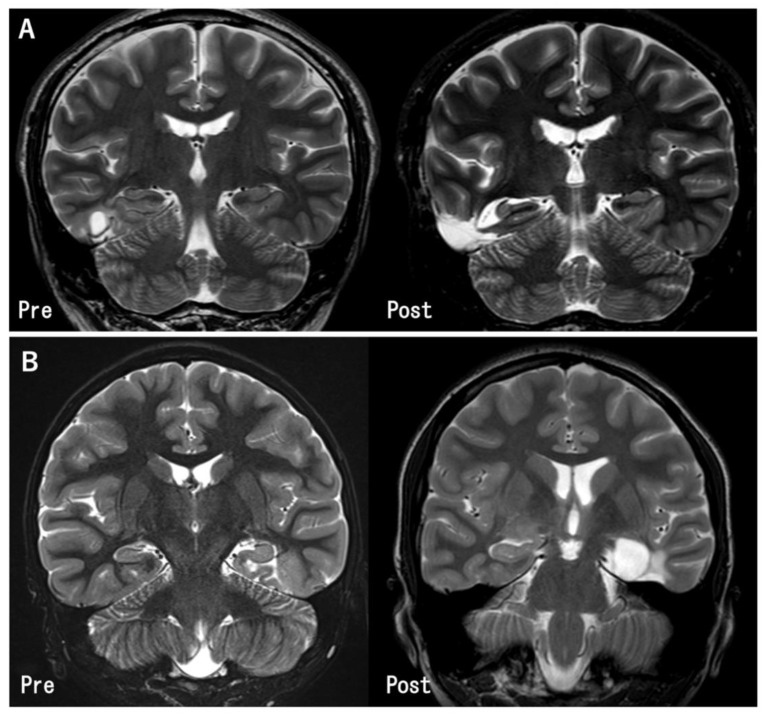
Pre- and post-operative MR images in the representative cases. (**A**) The tumor in the inferior temporal gyrus was totally removed. The hippocampus was preserved because it was located apart from the tumor (Case 11). (**B**) The tumor was located at the left parahippocampal gyrus. Gross total tumor removal and hippocampectomy was performed (Case 27).

Meanwhile, the hippocampus was preserved in 18 patients. The reasons for preserving the hippocampus were “distant tumor location from hippocampus” in 10 patients, “no preoperative cognitive decline” in 3 patients, “absence of abnormal epileptiform discharges on intraoperative ECoG” in 1 patient, and “unidentified” in 4 patients (Table 2 and Figure 2).

### 3.3. Effects of Hippocampal Resection

#### 3.3.1. Seizure Outcome

A total of 28 patients (87.5%) remained seizure-free (ILAE class 1) at the last follow-up, including 24 (75.0%) who were seizure-free after surgery (class 1a). Two patients achieved a class 3 outcome, one achieved a class 4 outcome, and one achieved a class 5 outcome. Twelve (85.7%) and sixteen (88.9%) patients achieved seizure freedom in the Resected and Preserved groups, respectively. The seizure-free rate was not statistically associated with hippocampal resection.

**Figure 3:**
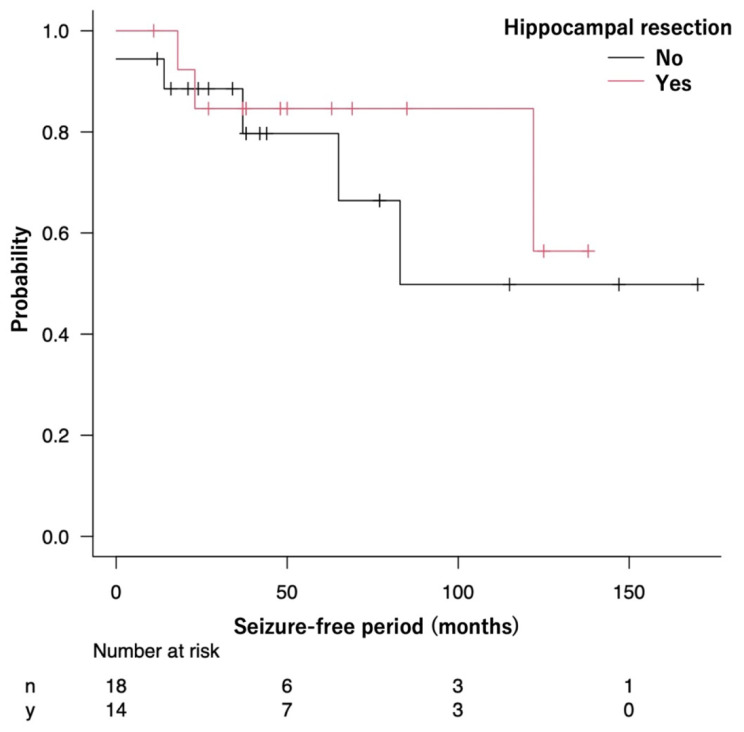
The Kaplan–Meier curve shows the seizure-free survival of patients in the Resected and Preserved groups.

The median SFP could not be calculated in the Resected group because more than half of the patients remained seizure-free. Meanwhile, the median SFP in the Preserved group was 83 months (95% CI: 37 months—not reached). The seizure-free survival curves were not significantly different between the Resected and Preserved groups (*p* = 0.52) (Figure 3).

The same analysis was performed in the limited patients with the tumor located medial to the collateral sulcus. The seizure-free survival curves were not significantly different between the two groups (*p* = 0.77) (Supplementary Figure S1).

#### 3.3.2. ASM Reduction

The number of postoperative ASMs was reduced in six (42.9%) and eight (44.4%) patients in the Resected and Preserved groups, respectively, but the difference was not significant. Meanwhile, ASMs were completely withdrawn in six patients (18.8%) (Table 2).

#### 3.3.3. Cognitive Outcome

**Figure 4:**
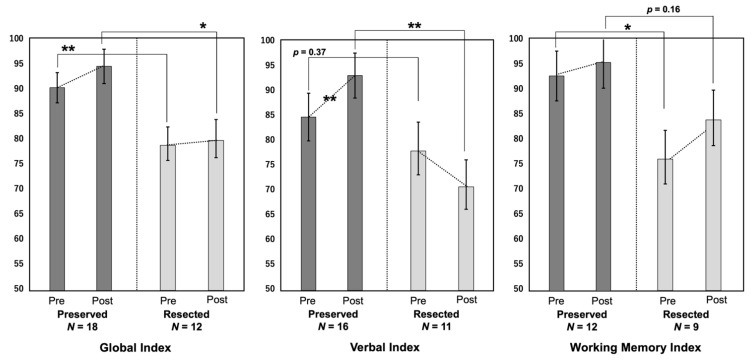
The pre- and postoperative scores in the three cognitive indices (global index, verbal index, and working memory index) are compared. *: *p* < 0.05, **: *p* < 0.01.

The IQ was evaluated with the WAIS in 15 patients, WISC in 11 patients, and Suzuki–Binet test in 1 patient. The DQ was evaluated with KIDS and Enjoji in four patients and one patient, respectively. The cognitive outcomes are summarized in Table 3 and Figure 4.

The preoperative GI and WMI and postoperative GI and VI were lower in the Resected group than in the Preserved group. The other indices also tended to be lower in the Resected group. The VI significantly improved postoperatively in the Preserved group (*p* < 0.01), whereas it tended to worsen in the Resected group, although the difference was not statistically significant (*p* = 0.31). The GI and WMI tended to improve after surgery in both groups, although the difference was not significant.

Controlling for the preoperative VI, ANCOVA revealed a statistically significant difference between the Resected and Preserved groups on the postoperative VI. The differences in the GI and WMI between both groups were not significant (Table 4).

## 4. Discussion

We investigated the influence of additional hippocampal resection on seizure and cognitive outcomes in a series of patients with temporal lobe LEATs and a radiologically normal hippocampus. The novelty of this study is that it only included patients with temporal LEATs and a normal hippocampus on MRI. Based on our results, additional hippocampal resection did not necessarily provide positive effects on seizure outcomes for patients with a normal hippocampus and instead had the potential of worsening verbal function.

Our results suggest that additional hippocampal resection has limited significance on seizure outcomes, further strengthening the evidence from previous reports. Research has shown that additional hippocampal resection does not increase the probability of seizure freedom [6,15,16]. Vogt et al. reported that hippocampal resection had no significant effect on postoperative seizure outcomes in a large cohort with temporal lobe LEATs [6]. Fried et al. reported that 87% of their 41 patients achieved seizure freedom from lesionectomy alone, and they did not find any association between the extent of hippocampal resection and seizure freedom [15]. In a study of 27 patients with temporal lobe LEAT performed by Morris et al., 81% achieved seizure freedom, but removing the mesial structures did not contribute to attaining seizure freedom [16]. These results suggest that the hippocampus should be preserved unless it has structural abnormalities. However, these retrospective studies included relatively small numbers of patients, and the number of patients with a normal hippocampus was not specified. An important issue that needs to be addressed is whether a structurally and functionally intact hippocampus should be resected; hence, our study strictly focused on patients without structural abnormalities in the hippocampus.

It may be reasonable to perform hippocampal resection to achieve better seizure outcomes when a hippocampal abnormality is present. An extensive review by Englot et al. reported that additional hippocampal resection significantly increased the seizure-free rate in patients with temporal lobe LEATs [4]. Cataltepe et al. and Mintzer et al. also supported the suitability of additional hippocampal resection [17,18]. However, these studies did not provide an answer for the issue we addressed because they did not exclusively include patients with a normal hippocampus. A few studies have proposed the efficacy of additional hippocampal resection in patients with a normal hippocampus. In a study of 15 paralimbic glioma patients without hippocampal invasion, Ghareeb et al. reported that additional hippocampal resection resulted in seizure freedom in all patients [7]. Morioka et al. performed hippocampal resection in patients without hippocampal invasion if the intraoperative electrocorticogram detected epileptic discharges and found pathological degeneration, such as neuronal loss or dysplastic neurons, in the resected hippocampus. The authors concluded that pathological abnormalities in the hippocampus supported the necessity of hippocampal resection [19]. In some cases, reoperation to remove the mesial temporal structures was necessary to control seizures after lesionectomy. In such cases, the hippocampus can be pathologically normal [16]. However, the studies mentioned above were based on small case series.

The indication for hippocampal resection may be based on the tumor’s location. In our study, most patients with a tumor lateral to the collateral sulcus underwent lesionectomy alone. In contrast, half of patients with a tumor located medial to the collateral sulcus underwent hippocampal resection. Yu et al. investigated temporal lobe epilepsy (TLE) patients with a normal hippocampus and concluded that the distance between the tumor and hippocampus was not a factor responsible for achieving seizure freedom [20]. The hippocampus is not necessarily epileptogenic, even if the tumor is located close to it. However, 96.9% of our patients showed impaired awareness during seizures, which is a typical sign of mesial TLE [21]. Hence, the hippocampus may be involved in seizures.

Intraoperative ECoG is commonly used to decide whether hippocampal resection should be performed. Sugano et al. proposed additional hippocampal resection based on the ECoG results because frequent spikes were observed in the hippocampus after resecting the lateral temporal lesions [22]. However, Yu et al. found that the intraoperative spikes and high-frequency oscillations of the hippocampus were not associated with postoperative seizure outcomes [20]. These data imply that intraoperative ECoG is not always reliable when determining whether the hippocampus is truly epileptogenic or not. A decision based on the intraoperative ECoG results should be carefully considered as it may lead to unnecessary surgical intervention.

The preoperative cognitive function was lower in the Resected group than in the Preserved group. A mesial tumor can easily affect hippocampal function more than a lateral tumor; therefore, the difference in the preoperative cognitive function between both groups can be attributed to the fact that 71.4% of patients in the Resected group had a tumor in the mesial temporal area. In addition to the VI, each cognitive index improved after surgery in the Resected group, although the changes were not significant. The results may imply that resection of a normal hippocampus has a negative impact on verbal function after surgery. Yu et al. described a significant improvement in intelligence, facial recognition, and logical memory after lesionectomy alone, and other cognitive functions showed no significant decline after surgery in patients with a normal hippocampus [20]. Previous studies including a small number of cases reported deterioration of memory in patients following lesionectomy on the dominant side [22,23]. Generally, hippocampal resection can worsen language and memory functions, especially following surgery on the dominant side [24]. Vogt et al. reported that patients with LEAT who underwent additional hippocampal resection showed significantly lower verbal memory scores than those who underwent lesionectomy alone [6]. The authors also reported that complete resection of the hippocampus resulted in lower verbal memory scores than partial resection. Wagner et al. reported that resection of the left-sided parahippocampal gyrus caused the decline in verbal learning performance even if the hippocampus was preserved [24]. The parahippocampal gyrus, a structure closely connected to the hippocampus, was resected together with the hippocampus in our patients with tumors in the parahippocampal gyrus. We suggest that, to achieve better functional outcomes, additional hippocampal resection should be carefully considered in patients with a normal hippocampus. A less invasive technique, such as multiple hippocampal transections, may also be a treatment option for lesions in the hippocampus.

This study had few limitations. The small sample size limited the statistical power of the study. The verbal memory score is a more appropriate index when considering the impact of resecting a normal dominant-sided hippocampus on cognitive function, although we had to use the VI and WMI to evaluate verbal and memory functions because of the limited number of patients who underwent the Wechsler Memory Scale test. This study possibly introduced selection bias due to its retrospective nature. The study was not designed to select patients randomly for hippocampal resection indication. The tumor location and preoperative cognitive function may have preoperatively influenced the indication of hippocampal resection. However, this study is novel in that it only included patients with a normal hippocampus, and the results may provide new evidence for surgical strategies for patients with temporal LEATs with the normal hippocampus.

## 5. Conclusions

In patients with temporal lobe LEATs, additional hippocampal resection is not necessary because lesionectomy alone results in good seizure control and additional hippocampal resection can adversely affect the postoperative language function. The results may provide a clue for establishing an optimal surgical strategy in these patients.

## Figures and Tables

**Table 1:** Patient characteristics.

Patients without Hippocampal Resection								
Case No.	Sex	Age (Years) at Epilepsy Onset (Mean: 10.2)	Age (Years) at Surgery (Mean: 21.6)	Follow-Up Period (Months)	Side of Surgery	Handedness	Seizure Classification	Seizure Frequency
1	M	14	22	92	L	R	FIAS	Monthly
2	M	6	26	34	L	R	FIAS > FBTCS	rare
3	F	17	55	38	L	R (corrected)	FIAS	Monthly
4	M	9	31	16	L	R	FIAS	Weekly
5	M	15	30	170	L	R	FIAS	Monthly
6	M	13	22	147	L	R	FIAS	Monthly
7	M	17	27	77	L	R	FIAS	rare
8	M	0	0.7	115	L	Unknown	FIAS	Weekly
9	M	12	13	12	L	L	FIAS	Monthly
10	F	18	32	30	L	R	FIAS	Weekly
11	M	4	27	27	R	R	FIAS	Weekly
12	F	15	17	24	R	R	FIAS	Weekly
13	F	9	15	64	R	R	FIAS	Monthly
14	F	13	14	21	R	R	FIAS	Weekly
15	M	10	23	16	R	R	FIAS	Weekly
16	M	8	29	44	R	R	FIAS	Weekly
17	M	0	1	160	R	Unknown	FIAS	Weekly
18	F	3	4	42	R	R	FIAS	rare
**Patients with hippocampal resection**								
**Case No.**	**Sex**	**Age (Years) at** **Epilepsy Onset** **(Mean: 5.4)**	**Age (Years) at** **Surgery** **(Mean: 14.6)**	**Follow-Up Period** **(Months)**	**Side of** **Surgery**	**Handedness**	**Seizure** **Classification**	**Seizure** **Frequency**
19	F	1	8	69	L	R	FIAS	Monthly
20	F	14	15	156	L	L	FIAS	Monthly
21	M	8	25	85	L	L	FIAS	Weekly
22	M	13	18	38	L	R	FIAS > FBTCS	rare
23	F	0	13	138	L	R	FIAS	Daily
24	M	10	10.8	127	L	R	FIAS	Weekly
25	M	0	0.5	125	L	Unknown	FIAS	Weekly
26	M	8	42	63	L	Ambiguous	FIAS	Monthly
27	F	4	7	37	L	R	FIAS	Weekly
28	F	3	6	50	L	R	FIAS	Weekly
29	F	0	9	59	R	R	FIAS + visual aura	Daily
30	F	1	1.8	27	R	Unknown	spasm	Daily
31	M	8	11	48	R	R	FIAS	Weekly
32	M	6	38	11	R	R	FIAS	Monthly

Abbreviations: F = female, FIAS = focal impaired awareness seizure, FBTCS = focal to bilateral tonic-clonic seizure, L = left, M = male, R = right.

**Table 2:** Surgery and surgical outcome.

Patients without Hippocampal Resection						
Case No.	Rationale of Hippocampal Preservation	Histology	ILAE Class	Follow-Up Duration (Months)	Number of Preoperative ASMs	Number of Postoperative ASMs
1	Unidentified	GNT/astrocytoma	3	92	3	3
2	Distant from hippocampus	GNT	1a	34	2	2
3	Distant from hippocampus	FCD + LGG	1a	38	3	2
4	Distant from hippocampus	LGG	3	16	6	5
5	Unidentified	DNT	1a	170	2	0
6	Distant from hippocampus	GG	1a	147	2	2
7	No preoperative cognitive decline	GG	1a	77	2	2
8	Unidentified	GG	1a	115	1	0
9	Distant from hippocampus	LGG	1a	12	1	1
10	Distant from hippocampus	PXA	1	30	1	1
11	Distant from hippocampus	GG	1a	27	3	1
12	Distant from hippocampus	GG	1a	24	2	2
13	Distant from hippocampus	GG	1	64	1	0
14	No preoperative cognitive decline	LGG	1a	21	1	0
15	No preoperative cognitive decline	GG	1a	16	2	2
16	Intraoperative ECoG findings	GG	1a	44	2	0
17	Unidentified	LGG	1	160	1	1
18	Distant from hippocampus	astrocytoma	1a	42	1	1
**Patients with Hippocampal Resection**						
**Case No.**	**Histology**		**ILAE Class**	**Follow-Up** **Duration** **(Months)**	**Number of** **Preoperative** **ASMs**	**Number of** **Postoperative** **ASMs**
19	LGG		1a	69	1	1
20	LGG		4	156	1	1
21	LGG		1a	85	1	1
22	LGG		1a	38	1	1
23	GG		1a	138	3	1
24	GG		5	127	2	3
25	GG		1a	125	2	1
26	GG		1a	63	2	2
27	FCD + LGG		1a	37	2	1
28	GG		1a	50	2	1
29	GG		1	59	3	1
30	astrocytoma		1a	27	1	0
31	GG		1a	48	1	1
32	LGG		1a	11	1	1

Abbreviations: ASM = anti-seizure medication, DNT = dysembryoplastic neuroepithelial tumor, FCD = focal cortical dysplasia, GG = ganglioglioma, GNT = glioneuronal tumor, ILAE = International League Against Epilepsy, LGG = low-grade glioma, PXA = pleomorphic xanthoastrocytoma.

**Table 3:** Pre- and postoperative cognitive outcomes.

	Preoperative Score (Mean ± SE)			Postoperative Score (Mean ± SE)		
	GI	VI	WMI	GI	VI	WMI
Overall (*n* = 32)	85.7 ± 2.5	81.9 ± 3.7	85.6 ± 4.0	88.7 ± 2.9	83.9 ±4.0	90.5 ± 3.9
Preserved group (*n* = 18)	90.3 ± 3.0	84.7 ± 4.8	92.8 ± 4.9	94.6 ± 3.4	93.0 ± 4.5	95.4 ± 5.1
Resected group (*n* = 14)	76.8 ± 3.7	77.9 ± 5.8	76.1 ± 5.7	79.8 ± 4.2	70.7 ± 5.4	83.9 ± 5.9

Abbreviations: GI = global index, SE = standard error, VI = verbal index, WMI = working memory index.

**Table 4:** Analysis of covariance for the effect of hippocampal resection on postoperative cognitive index.

		Estimate	Standard Error	F Value	T Value	*p* Value
GI	Intercept	22.22	13.87		1.60	0.12
(*n* = 30)	Pre-GI	0.77	0.16	22.41	4.73	<0.0001
	HR	2.97	2.23	1.76	1.33	0.20
VI	Intercept	36.15	12.83		2.82	0.01
(*n* = 27)	Pre-VI	0.56	0.15	13.37	3.66	0.001
	HR	9.23	2.92	9.98	3.16	0.004
WMI	Intercept	27.92	14.82		1.88	0.08
(*n* = 21)	Pre-WMI	0.73	0.17	18.01	4.24	<0.001
	HR	−0.32	3.18	0.01	−0.10	0.92

Abbreviations: GI = global index, HR = hippocampal resection, VI = verbal index, WMI = working memory index.

## Data Availability

Not applicable.

## Supplementary Materials

The following supporting information can be downloaded at: https://www.mdpi.com/article/10.3390/brainsci12101381/s1, Figure S1: The Kaplan–Meier curve shows the seizure-free survival of patients in the Resected and Preserved groups in the limited patients with the tumor located medial to the collateral sulcus.
