# Supplementary material for: Is Hippocampal Resection Necessary for Low-Grade Epilepsy-Associated Tumors in the Temporal Lobe?
Source: Brain Sci. 2022 Oct 12;12(10):1381. doi: 10.3390/brainsci12101381 (PMC9599302; doi:10.3390/brainsci12101381)
Supplement: Supplementary file 1 [file brainsci-12-01381-s001.zip › brainsci-1944977-supplementary.pdf]

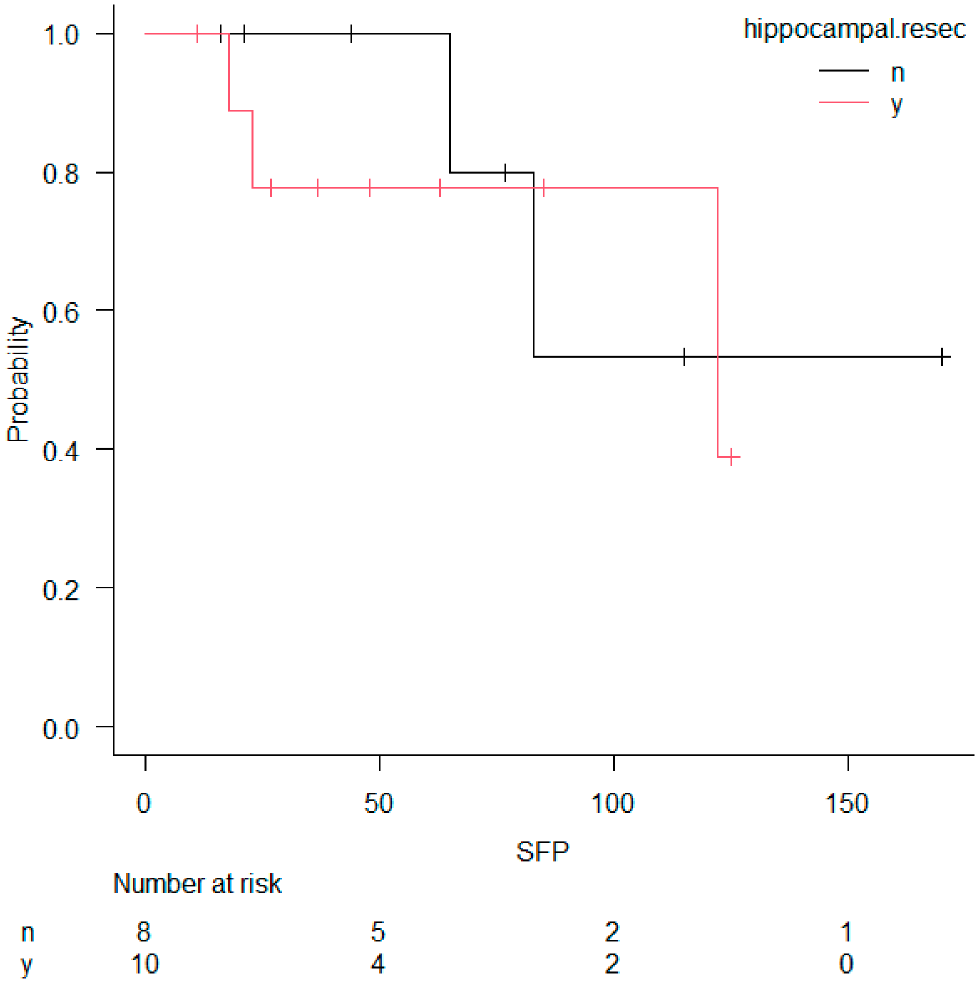

Figure S1. The Kaplan–Meier curve shows the seizure-free survival of patients in the Resected and Preserved groups in the limited patients with the tumor located medial to the collateral sulcus.
